# High-value care for critically ill oncohematological patients: what do we know thus far?

**DOI:** 10.5935/2965-2774.20230405-en

**Published:** 2023

**Authors:** Fernanda Chohfi Atallah, Pedro Caruso, Antonio Paulo Nassar Junior, Andre Peretti Torelly, Cristina Prata Amendola, Jorge Ibrain Figueira Salluh, Thiago Gomes Romano

**Affiliations:** 1 Discipline of Anaesthesiology, Pain and Intensive Care, Escola Paulista de Medicina, Universidade Federal de São Paulo - São Paulo (SP), Brazil; 2 AC Camargo Cancer Center - São Paulo (SP), Brazil; 3 Hospital Santa Rita - Santa Casa de Misericórdia de Porto Alegre - Porto Alegre (RS), Brazil; 4 Hospital de Câncer de Barretos - Barretos (SP), Brazil; 5 Instituto D’Or de Pesquisa e Ensino - Rio de Janeiro (RJ), Brazil; 6 Instituto D’Or de Pesquisa e Ensino - São Paulo (SP), Brazil

**Keywords:** Neoplasms, Low-value care, Cost of illness, Hospital costs, Critical illness, Patient care management, Intensive care units

## Abstract

The number of patients with cancer requiring intensive care unit admission is increasing around the world. The improvement in the pathophysiological understanding of this group of patients, as well as the increasingly better and more targeted treatment options for their underlying disease, has led to a significant increase in their survival over the past three decades. Within the organizational concepts, it is necessary to know what adds value in the care of critical oncohematological patients. Practices in medicine that do not benefit patients and possibly cause harm are called low-value practices, while high-value practices are defined as high-quality care at relatively low cost. In this article, we discuss ten domains with high-value evidence in the care of cancer patients: (1) intensive care unit admission policies; (2) intensive care unit organization; (3) etiological investigation of hypoxemia; (4) management of acute respiratory failure; (5) management of febrile neutropenia; (6) urgent chemotherapy treatment in critically ill patients; (7) patient and family experience; (8) palliative care; (9) care of intensive care unit staff; and (10) long-term impact of critical disease on the cancer population. The disclosure of such policies is expected to have the potential to change health care standards. We understand that it is a lengthy process, and initiatives such as this paper are one of the first steps in raising awareness and beginning a discussion about high-value care in various health scenarios.

## INTRODUCTION

Cancer mortality has decreased in the past three decades;^(1)^ however, the number of cancer patients requiring intensive care unit (ICU) admission is increasing. Data suggest that 25% to 30% of the beds in ICUs are occupied by cancer patients and that cancer characteristics are not associated with worse outcomes in the short term.^(2)^

The evolving knowledge of critically ill patients with cancer has introduced new concepts in patient management and ICU admission policies.^(3,4)^ Within organizational concepts, it is necessary to know what adds value in caring for critical oncohematological patients; thus, the term “high-value care” was coined.

High-value care practices are defined as high quality of care at relatively low cost.^(5)^ To identify what these practices are, researchers examine “positive deviants” (i.e., practices with good results associated with better outcomes;^(6)^ practices that already exist; and practices that health care providers with expertise can generalize).^(7,8)^ The application of these practices has already been successfully applied in child nutrition and gestational care.^(9,10)^

It is known, however, that the mere publication of items with scientific evidence of low-value care reduction alone will not be effective. Change will come from a broader discussion of culture, the benefit of economic savings in cutting low-value care, and the involvement of sectors of society so that knowledge of these topics will not be limited to the medical community.^(11)^ Cliff et al. demonstrated that the disclosure of such items has the potential to change patterns of health activity.^(12)^

Decreasing low-value care has the potential for cost savings; therefore, discussion of the best payment models is of paramount importance. It is logical to think that the fee-for-service model, in which payment is based on the procedure executed, stimulates low-value care practices when compared to other models such as fee-for-performance.^(13,14)^ The above statement has a rationale, but although a cross-sectional study by Park et al. found that 13 low-value services were similarly present in two different models, practices such as unnecessary cancer screening and antibiotics for upper respiratory infection were as prevalent in a fee-for-service model as in a fee-for performance model.^(13)^ This suggests that the model is one of the topics that should be addressed to cut low-value care costs, along with health service costs, education, protocol development and better career plans for health care workers.^(14)^

**Figure 1:**
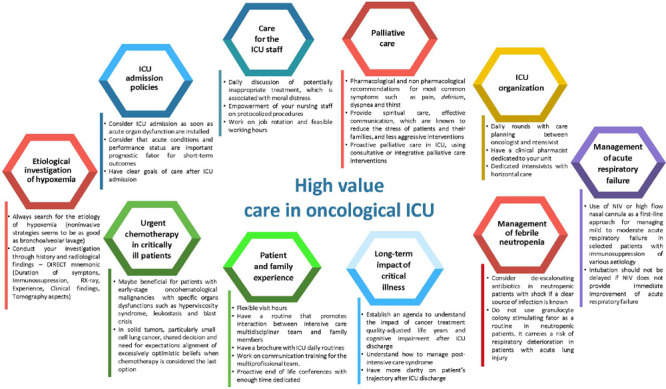
High-value care in the oncological intensive care unit

We know that it is a lengthy process, and in this article, through the collaboration of seven experts on oncologic critical care, we list 10 domains with evidence of high value in the care of patients with cancer (Figure 1).

ICU - intensive care unit; NIV - noninvasive ventilation.

### 1. Intensive care unit admission policies

Intensive care unit admission of patients with cancer is frequently delayed,^(15,16)^ but it is known that delayed admission and oxygen therapy use are associated with higher mortality,^(17)^ while rapid ICU transfer is associated with better outcomes.^(18)^ Therefore, timely ICU admission is a measure of high-value care. Lengline et al. demonstrated that in patients with acute myeloid leukemia and a high risk of tumor lysis, direct admission to the ICU, even if there was no organ dysfunction, was associated with better outcomes.^(19)^ Applying objective criteria, such as the modified early warning score (MEWS), can be a strategy to improve ICU admission at the right time.^(20)^

It is of paramount importance to realize that cancer-related characteristics do not predict short-term outcomes. Actually, the severity of organ dysfunction at ICU admission and performance status are more sensitive prognostic predictors. ^(15,21,22)^

In cases of uncertainties related to the benefit of ICU “full code care”, a time-limited trial (TLT) is a high-value care practice.^(23,24)^ Lecuyer et al., in a pioneering study about TLT, demonstrated that the behavior of organ dysfunction up to the fifth day of ICU admission accurately predicted ICU mortality in nonbedridden patients with cancer. In patients requiring mechanical ventilation, vasopressors or renal replacement therapy, 3 days of treatment was enough to predict ICU mortality.^(25)^

The question of the TLT duration may have some peculiarities. Shrime et al. showed that a TLT of up to 4 days for patients with solid tumors and more severe organ dysfunction could discriminate 30-day survival. However, Shrime et al. concluded that for patients with hematological malignancies or with lower levels of organ dysfunction, a longer TLT duration, up to 2 weeks, is recommended.^(26)^

Regardless of the nuances of the evolution of each patient, it is important that we have a clear objective for admitting patients with cancer to the ICU. We know that some symptom control conditions require resources that the ward cannot provide; therefore, some patients can be admitted for symptom care.^(27)^

In conclusion, points of ICU admission high-value care are as follows: admission as soon as organ dysfunction is identified; breaking the paradigm that cancer characteristics alone are prognostic factors; and TLT use in cases with uncertainties about the short-term evolution.

### 2. Intensive care unit organization

Intensive care unit practices and team organization have an impact on the outcome of patients with cancer. Hawari et al. demonstrated that implementing a high-intensity ICU staffing model was associated with a decrease in mortality by 15% to 60% in critically ill patients with cancer.^(28)^ Additionally, Soares et al. showed that the presence of clinical pharmacists in ICUs was associated with increased odds of survival in critically ill patients with cancer.^(29)^

Effective communication and therapeutic planning are essential in an oncological ICU because there may be points of care that are unknown by the ICU team, such as chemotherapy support, particularities of cancer care and alignment of patient and family expectations. However, oncologists and intensivists have different knowledge, and conflicts may arise about the proper management of these patients. A survey was conducted in Brazil among oncologists and intensivists at 2 academic cancer centers on the management of hypothetical patients with different types of cancer (metastatic pancreatic cancer and metastatic breast cancer) who developed septic shock and multiple organ failure.^(30)^ The results showed that although most oncologists and intensivists were in agreement about the goals of treatment, there were significant differences in how they approached the management of these two hypothetical cases. Intensivists favored the withdrawal of life support measures for patients with breast cancer more than oncologists did (54% *versus* 21%; p &lt; 0.001). The results of this study suggested that oncologists tend to focus on cancer characteristics, while intensivists focus on multiple organ failure. Regular meetings between oncologists and intensivists can reduce potential conflicts regarding the intensive care of patients with cancer.^(30)^ Therefore, this integration is very important and is the rationale behind why daily rounds are associated with reduced hospital mortality and a more efficient use of resources in critically ill patients with cancer.^(31)^

In a retrospective analysis of 129,680 admissions in 93 ICUs, Zampieri et al. characterized the ICUs into three “phenotypes” based on three organizational characteristics: degree of nursing autonomy (measured by a score of autonomy in domains such as drug-drug titration, sedation and nutrition, active mobilization, weaning from mechanical ventilation and medication to control symptoms), presence of a dedicated clinical pharmacist, and presence of intensivists with certification 24 hours/7 days a week.^(32)^ Their study suggested that patients treated in ICUs combining expert intensivist coverage, a dedicated pharmacist and nurses with greater autonomy had the best outcomes.^(32)^

Notably, the number of available protocols for the prevention of care-related infections was also significantly different in ICUs with better performance, with a higher average in ICUs with better performance.^(32)^

In conclusion, high-intensity ICU staffing models, with the presence of board-certified intensivists 24 hours/7 days a week, a dedicated clinical pharmacist, daily rounds in the ICU with the presence of an oncologist, and a higher degree of nursing autonomy in domains preestablished by protocols, are part of what we currently interpret as positive deviants of high-value care.

### 3. Etiological investigation of hypoxemia

The main cause of ICU admission of patients with cancer is hypoxemic respiratory failure.^(33)^ Cancer patients with hypoxemic respiratory failure require immediate empirical treatment, but it is also of paramount importance to reach the cause of respiratory failure, since patients without an etiological diagnosis have a higher mortality.^(34)^

The etiological investigation of hypoxemic respiratory failure depends on the arsenal of complementary tests available and the care team’s expertise. The most striking change in the investigation was the introduction of molecular biology techniques and biomarkers that allow etiological diagnosis in approximately 80% of patients with or without cancer.^(4,35)^ Studies using a systematic approach and a robust diagnostic arsenal have shown that approximately two-thirds of the causes of hypoxemia are infectious, while one-third are divided between noninfectious or indeterminable causes (Table 1).^(36)^

**Table 1:** Etiological diagnoses most frequently reported in studies of patients with cancer and hypoxemic respiratory failure

Study	Infectious diagnosis	Noninfectious diagnosis	Without diagnosis
Azoulay et al.^(4)^*	**Total 69.1%**	Total 15.9% *	**20.3%**
	Bacteria 41.6%	Tumor infiltration 8.8%	
	Virus 6.2%	Cardiogenic edema 6.2%	
	Yeast 12.4%	Organizing pneumonia 0.9%	
	Pneumocystis 8.0%		
	Toxoplasmosis 0.9%		
Wohlfarth et al.^(35)^†	**Total 71%**		**29%**
	Bacteria 11.5%		
	Virus 16.7%		
	Yeast 17.9%		
	Polymicrobial 12.8%		
	Others 12.1 %		
Yoo et al.^(36)^	**Total 64%**	**Total 23%**	**13%**
	Bacteria 29%	Tumor infiltration 6%	
	Virus 18%	Pneumonitis by drug 6%	
	Yeast 9%	Cardiogenic edema 5%	
	Pneumocystis 7%	Alveolar hemorrhage 4%	
	Tuberculosis 1%	Others 2%	

* In the study by Azoulay et al.^(4)^, 8% of patients had more than two diagnoses, † The Wohlfarth et al.^(35)^ study exclusively evaluated patients after allogeneic bone marrow transplantation. The repertoire of tests includes only microbiological tests.

The systematic approach begins with clinical investigation, and the *Direct* mnemonic described in the study by Schnell et al. is a relevant aid.^(37)^ Imaging tests are indispensable because they narrow the diagnostic hypotheses but are insufficient because they usually do not allow etiological diagnosis. To collect samples of lower airway secretions, it is possible to use an invasive approach, via bronchoscopy, or a noninvasive approach. Although the invasive approach seems more productive, there is at least one randomized study showing that the noninvasive strategy is equally effective when compared to the use of bronchoscopy with bronchoalveolar lavage.^(4)^

Finally, when empirical treatment does not yield a positive result and a noninvasive or minimally invasive approach does not allow etiological diagnosis, an invasive approach, which consists of transbronchial or open lung biopsy, should be considered. Due to the high risk of pneumothorax or bleeding, transbronchial biopsy is usually contraindicated in patients with thrombocytopenia or with positive pressure ventilation.^(38,39)^ In patients without thrombocytopenia and outside positive pressure ventilation, the safety of transbronchial biopsy is greater, and the diagnostic gain is high for diseases with peribronchial involvement. As the diagnostic gain for infectious diseases is greater for bronchoalveolar lavage than for lung biopsy, there is no point in replacing bronchoalveolar lavage with transbronchial biopsy, but they can be considered complementary.^(39)^ In a study with non-HIV immunocompromised patients, the combined diagnostic gain of bronchoalveolar lavage with transbronchial biopsy was greater than the gain of bronchoalveolar lavage alone.^(40)^

Open lung biopsy is an exception for etiological diagnosis. The ideal time to indicate an open biopsy is uncertain, but biopsies performed immediately after the onset of hypoxemia are not superior to noninvasive or minimally invasive methods, while biopsies performed more than 10 days after recognition of the pulmonary infiltrate do not decrease in-hospital mortality.^(41)^ The mortality of an open lung biopsy is not higher than that of most elective surgeries, occurs in approximately 2% of cases, and is almost exclusively caused by bleeding and hemothorax. The incidence and occurrence of severe bleeding are associated with coagulation disorders, especially thrombocytopenia.^(42)^ Compared to bronchoalveolar lavage, open lung biopsy has a higher gain for noninfectious diagnoses but a lower gain for infectious diagnoses with a higher probability of complications.^(40)^

### 4. Management of acute respiratory failure

Acute respiratory failure (ARF) is the main cause of unplanned ICU admission among patients with cancer. ^(43,44)^ The most common etiology of ARF in these patients is lung infection, which represents approximately 65% of patients with acute respiratory distress syndrome (ARDS).^(45)^ The risk of ARF is higher in patients with hematological malignancies than in those with solid tumors, especially in patients with neutropenia and those undergoing bone marrow transplantation.^(46,47)^ Patients with cancer and febrile neutropenia may develop a distinct form of ARDS that occurs during the neutrophil recovery phase in association with the administration of granulocyte colony stimulating factor (G-CSF).^(48,49)^ Lung cancer is the most frequent tumor associated with respiratory complications among patients with solid tumors.^(50)^ Other frequent causes of ARF are drug-related pulmonary toxicity, radiation and clinical situations frequently presented in patients with cancer, such as chronic obstructive pulmonary disease, cardiogenic pulmonary edema, diffuse alveolar hemorrhage and acute lung injury associated with transfusion (TRALI). In patients affected by acute leukemia, ARF may be caused by leukemic pulmonary infiltration, leukostasis and pneumopathy secondary to tumor lysis.^(51)^

The hospital mortality of patients with cancer and ARF is approximately 50%, depending on the etiology, severity, need for invasive mechanical ventilation (IMV) and associated organ dysfunction.^(52)^ A multicenter, prospective, observational study evaluated 1,611 immunosuppressed patients (52% oncohematological and 35% with solid tumors) with ARF admitted to 68 ICUs between 2015 and 2016. Among the 1,611 patients analyzed, 596 patients (37%) were intubated at ICU admission or in the emergency room, and of these, 52% died. A total of 915 (56.8%) patients received noninvasive support first, such as standard oxygen therapy, high-flow nasal cannula (HFNC), noninvasive ventilation (NIV) or combination NIV plus HFNC. Approximately 40% of these patients required tracheal intubation during hospitalization. Approximately 85% of patients who failed noninvasive strategies and required IMV died.^(52)^

The best initial strategy for the ventilatory management of oncological patients with ARF still raises doubts and uncertainties. Previous studies have suggested that early NIV could improve survival and reduce the incidence of intubation and IMV.^(53,54,55,56)^ In 2001, Hilbert et al. compared the use of NIV with standard oxygen therapy in a randomized controlled trial that included 54 immunosuppressed patients (58% oncohematological) with fever, pulmonary infiltrate and hypoxemic ARF. Patients in the NIV group had a lower intubation rate (46% *versus* 77%) and hospital mortality compared to the group with standard oxygen therapy (50% *versus* 81%).^(54)^ Recent studies did not confirm previous reports of early NIV benefits compared to oxygen therapy. ^(57,58)^ A multicenter, randomized, prospective study conducted in 28 hospitals in France and Belgium evaluated the best ventilatory strategy (NIV *versus* oxygen mask) in 374 immunosuppressed patients with hypoxemic ARF. Neither 28-day mortality nor the intubation rate differed between the groups.^(58)^

Noninvasive ventilation failure is more frequent in patients with cancer than in the general population and is associated with more complications related to intubation and worse outcomes.^(59,60,61,62,63,64)^ Some identified factors associated with NIV failure in previous studies are a high respiratory rate, the time interval between ICU admission and NIV initiation, the need for vasopressor or renal replacement therapy and the ratio of partial pressure of oxygen and fraction of inspired oxygen (PaO_2_/FiO_2_) &lt; 200.^(59,62,65)^ Although there are no specific data available on patients with cancer, NIV can be considered in patients with cardiogenic pulmonary edema or exacerbation of chronic obstructive pulmonary disease with respiratory acidosis.^(66)^ However, intubation should not be delayed if NIV does not provide immediate improvement of ARF.^(66)^

High-flow nasal cannula appears to be a promising therapy for patients with cancer. In a retrospective cohort of 178 patients with ARF, 76 (43%) received NIV plus HFNC, 74 (42%) received NIV plus standard oxygen therapy, 20 (11%) received only HFNC and 8 (4%) received only standard oxygen therapy. The combination of NIV and HFNC was associated with lower mortality rates (37% *versus* 52%, p = 0.04) and higher survival at 28 days.^(67)^ Two randomized studies that evaluated HFNC in immunosuppressed patients were recently published.^(68,69)^ The HIGH study was a multicenter, prospective study that randomized 778 immunosuppressed patients with ARF into two groups: HFNC and oxygen therapy. The primary outcome was 28-day mortality. Mortality between groups was similar (35.6% *versus* 36.1%), as was the percentage of patients who required IMV (38.1% *versus* 43.8%).^(68)^ The FLORALI-IM study published in 2022 evaluated the use of HFNC *versus* NIV intercalated with HFNC in 299 immunosuppressed patients with ARF. There was no difference in 28-day mortality, intubation or IMV use.^(69)^ There are no studies demonstrating the superiority of HFNC therapy over NIV or standard oxygen therapy in patients with cancer and ARF. However, hospital mortality in patients with cancer has been decreasing over the years, probably due to better ARF approaches, including etiological investigation, early ICU admission and protective ventilation.

### 5. Management of febrile neutropenia

In febrile neutropenic patients, the first dose of antibiotic should be administered within the first hour after blood culture collection.^(70)^ Fever in neutropenic patients should be considered a medical emergency.

Empirical antimicrobial therapy must broadly cover the most likely pathogens, either guided by suspected infections or epidemiological features. Gram-negative bacteria are the main cause of infections in sites outside of the bloodstream; therefore, gram-negative pathogens are the first target of antimicrobial coverage.^(71)^

Treatment for Gram-positive bacteria, which are the most frequent pathogens identified in neutropenic patients with fever, can be performed using vancomycin (or teicoplanin) in cases of suspected intravascular catheter infection, blood culture with gram-positive bacteria in identification, hemodynamic instability, soft tissue or skin infections or mucositis grade &gt; l.^(72)^

In patients without gram-positive bacteria, empirical antibiotic therapy should be discontinued within 48 to 72 hours. In patients colonized or suspected with vancomycin-resistant enterococcus, empirical treatment should be guided by the following criteria: hematological or solid tumors colonized by vancomycin-resistant enterococcus associated with clinical stability; persistence of fever and neutropenia with the use of carbapenem plus vancomycin for more than 72 hours; or clinical instability.^(73,74)^

Usually, fungi are not the initial cause of fever in neutropenic patients, and initial empirical coverage is indicated only in patients with invasive fungal disease (histological or microbiological findings); after ≥ 4 days of persistent fever, if the radiological pattern is compatible with fungal disease; or if febrile neutropenia persists for more than 7 days. ^(73)^ The recommendations of the guidelines of the International Disease Society of America (IDSA) suggest the use of caspofungin (70mg on the first day followed by 50mg/day),) and amphotericin B liposomal as the second choice (3 - 5mg/kg/day). The third option in stable patients without prior imidazole use is fluconazole (800mg of attack, followed by 400mg/day).^(75)^ If aspergillosis is a strong clinical hypothesis or the patient is using echinocandins for prophylaxis, liposomal amphotericin B should be the first choice.^(75)^ Micafungin and anidulafungin have not been adequately tested in patients with febrile neutropenia; however, they can be used as an alternative in the absence of caspofungin since the spectrum and antifungal activity of the two agents are similar.^(76)^

The decision on antimicrobial maintenance should be reviewed within 72 and 96 hours (Table 2).

**Table 2:** Duration of antibiotic treatment

Situation	Treatment time
Afebrile, no defined focus, with response to initial antimicrobial regimen	48 hours afebrile if neutrophils above 500 cells/mm³
Afebrile, without a defined focus, with response to modified treatment	7 days afebrile if neutrophils less than 500 cells/mm³
Afebrile and with a defined focus	Suggested time for the site in question
Infection of skin and soft tissues	Duration of treatment: 7-14 days
Bloodstream infection	*Gram*-negative: 10-14 days
	Coagulase-negative *Staphylococcus:* 7 days
	*Staphylococcus aureus:* 14 days + transesophageal echo
	Candida sp.: 14 days after negative blood culture + transesophageal echo
Catheter-related infection	Remove catheter if infection by *Staphylococcus aureus, Candida* Sp or tunnel infection
Bacterial pneumonia	Duration of treatment: 7 days
Diarrhea due to *Clostridioides difficile*	Duration of treatment: 10-14 days

Granulocytic stimulating agents can be used in patients without response to antimicrobial treatment in the presence of sepsis or septic shock; outside this situation, the use should be individualized.^(77)^

Regarding prevention, the most effective measure is hand hygiene, especially during the management of neutropenic patients.^(78)^ There is no benefit of specific protective measures to neutropenic patients, such as masks, gloves or aprons, but all patients should be subjected to standard precautions, such as hand hygiene (washing hands with soap and water for 2 minutes); appropriate use of personal protective equipment; respiratory hygiene; careful handling of materials, equipment, clothing and food utensils; environmental hygiene; prevention of accidents with sharps and biological materials; safe practice in the preparation and administration of medications; exclusion of plants and flowers in rooms or units of neutropenic patients; and contraindication of rectal manipulation in neutropenic patients (anal swab, thermometers, enemas, etc.).^(79)^ Three meta-analyses did not recommend the use of specific diets for neutropenic patients as a measure for the prevention of infection; however, a well-cooked diet without raw food was suggested for all neutropenic patients.^(80)^

### 6. Urgent chemotherapy treatment in critically ill patients

The use of chemotherapy in patients admitted to the ICU is an exception. Few tumors have a rapid response to chemotherapy to the point of reversal of organ dysfunction caused by the cancer itself. The studies that have evaluated the use of chemotherapy in the ICU were small, observational, retrospective, and single center. In general, chemotherapy in the ICU is acceptable in patients with hematological malignancies with complications leading to organ dysfunction, such as hyperviscosity syndrome, leukostasis and blast crisis.^(81)^ In patients with solid tumors, chemotherapy in the ICU is often indicated in involvement, such as acute liver failure secondary to liver metastases, malignant tumor obstructions caused by tumors of the gastrointestinal tract and acute respiratory failure secondary to bronchial obstruction in lung tumors. However, with the exception of patients with small cell lung carcinomas and some germ-cell tumors associated with complications, the use of emergency chemotherapy in the ICU for solid tumors is associated with higher mortality.^(82,83,84,85)^

### 7. Patient and family experience

The World Health Organization (WHO) considers that patient experience involves the perspectives of patients, families, and communities, considering them as participants and beneficiaries.

Relatives of patients who experience a critical illness have more symptoms associated with anxiety, depression and posttraumatic stress than patients themselves,^(86)^ and simple measures such as time devoted to communication, with proactive end-of-life conferences and a printed copy of the unit’s guidelines, can help reduce such symptoms.^(87)^ Interactions between intensivists, other physicians and family members are other means of caring for such symptoms, with minimal costs but significant impact. Family meetings are the cornerstone of facilitating open communication, adhering to the care plan and minimizing distress between family members and health care providers.^(88,89)^

Although the time of interaction is an important factor, with a rationale for better disease understanding, participation in the decision-making process and interaction with the ICU staff, extending visiting hours remains controversial in the literature. The ICU Visits Group Investigators showed that an extended visiting hours policy compared to a more restrictive one (4.8 *versus* 1.4 hours) did not reduce delirium; however, there was a decrease in anxiety and depression among family members.^(90)^ Regardless of the outcome, the preparation of the multidisciplinary team to treat these families is imperative.

### 8. Palliative care

According to the WHO, in a concept defined in 1990 and updated in 2002, “Palliative Care consists of assistance provided by a multidisciplinary team, which aims to improve the quality of life of patients and their families, in the face of a disease that threatens life, through the prevention and relief of suffering, through early identification, impeccable assessment and treatment of pain and other physical, social, psychological and spiritual symptoms”.

The role of palliative care for patients with cancer in the ICU is extremely important. These patients frequently present physical, psychosocial and spiritual suffering. Thus, the integration of palliative care in this scenario is associated with better quality of life for patients and families, more occurrence of advance directives and decreased use of nonbeneficial interventions to prolong life.

The symptoms most commonly presented by patients with cancer are pain, delirium, dyspnea and thirst, and palliative care support, particularly in pain management, has been proven to have a positive impact.^(91,92)^ A retrospective study evaluated 1,383 admissions to an oncological ICU, where 88 patients were evaluated by the palliative care team, and several opportunities for care improvement were identified. Additionally, the palliative care team made numerous pharmacological and nonpharmacological recommendations alleviating distressing symptoms and increasing do not resuscitate orders and withdrawal of IMV and noninvasive mechanical ventilation.^(92)^ The provision of spiritual care and effective communication also contribute to reducing patient and family stress, leading to less aggressive interventions and more appropriate hospice indications. Finally, evidence suggests that proactive palliative care in the ICU, using consultative or integrative palliative care interventions, shortens hospital and ICU length of stay^(93)^

In conclusion, there is no doubt that palliative care in the ICU has numerous benefits to patients with cancer; however, challenges remain in terms of greater integration, additional training of intensivists in skills such as communication and symptom control of end-of-life patients, and earlier initiation of palliative care within the ICU.

### 9. Care of intensive care unit staff

One crucial point in the evaluation of the ICU employee experience is the development of burnout. Fumis et al. demonstrated that moral distress due to therapeutic obstinacy and futility in treatment are the main risk factors for moral distress.^(94)^

The priority of ICU managers should be ICU efficiency in achieving its clinical goals while maintaining a humanized environment. Despite the excessive emphasis on the technological aspects of intensive care, it is the “human factor” that mediates the results of an ICU.^(95)^ Additionally, organizational aspects such as ICU strain, suboptimal staffing patterns and lack of ICU resources have a significant burden on the ICU team.^(96)^ In this sense, the COVID-19 pandemic was a scenario where all the abovementioned factors were simultaneously present for a long period of time, and studies have demonstrated that this adversely affected the well-being of health care personnel.^(97)^

The volume and timing of work, particularly the number of nights and consecutive days worked, appear to be significant factors determining the chance of burnout among intensivists, which is associated with a desire to quit their jobs.^(98)^ Strategies to reduce the ICU workload and decrease the burnout rate in multidisciplinary ICU teams are needed. Reducing the workload, avoiding shifts longer than 12 hours, and restricting the maximum number of consecutive days worked, including paid vacations, psychological support, and well-compensated pay, are viable approaches for preventing burnout in professionals.^(99)^

Oncologic critical care is a highly complex scenario where a combination of frequent discussions around end-of-life care, a high workload, and inadequate communication (as well as inter- and intrateam conflicts) generates stress and burnout in up to half of the professionals.^(100)^ Therefore, we propose the use of known risk factors for moral distress and burnout as targets for quality improvement, aiming to reduce their prevalence and therefore burnout.

From the organizational perspective, it is vital to guarantee that an ICU is well staffed and resourced but also that it is a resilient ICU and therefore capable of adequately responding to daily challenges as well as unexpected situations (i.e., catastrophes, pandemics).^(101)^

### 10. Long-term impact of critical disease on the cancer population

Post-intensive care syndrome as well as other long-term debilitating conditions are often described in survivors of severe and prolonged critical illness.^(102,103)^ Although the short-term mortality of ICU patients with cancer has substantially improved over the last few decades,^(104,105)^ patient-centered outcomes and long-term survival remain suboptimal.^(106)^ Patients with leukemia requiring ICU admission, relapsed or refractory disease, secondary leukemia, or multiorgan failure therapy were independently associated with 1-year mortality.^(107)^ These factors associated with adverse long-term outcomes are similar to those observed in patients with solid tumors.

Regarding the quality of life of survivors, a study demonstrated that among the baseline poor quality of life risk factors, previous health-related quality of life and performance status were associated with better outcomes at the 18-month follow-up.^(106)^ In patients with hematologic malignancies, poor quality of life associated with impaired physical and mental health issues was observed at the 3-and 12-month follow-ups.^(108)^ Additionally, the challenges of ICU survivors are not restricted to their quality of life but also to the impact on the potential continuity of cancer treatment. Loss of functional capacity is often described in ICU survivors, especially those who experience prolonged ICU stays and mechanical ventilation. The fact that up to 80% of cancer patients discharged from the ICU may not receive optimal chemotherapy should alert clinicians and oncologists and guide them to engage early in the discussion of the goals of care, considering that ICU discharge will not guarantee cancer survival. (15) In a study of patients with lung cancer treated in the ICU, 38% of survivors had to change their initially planned treatment regimen.^(109)^

Among the priorities to ensure high-value care for ICU cancer patients, intensivists and oncologists should consider the expected quality of life of survivors as well as their ability to remain eligible for highly effective anticancer therapies. In addition, patients with cancer should be considered for rehabilitation after critical illness. Although specific evidence related to cancer patients is missing, physical and nutritional rehabilitation has been successfully tested in general ICU survivors.^(110)^

A combination of strategies aiming to improve muscle strength and endurance, respiratory function, nutrition and cognitive status is currently proposed to counteract the effects of postintensive care syndrome (PICS).^(111,112,113)^ This is of utmost importance in ICU survivors and may be even more relevant for patients with cancer, as the loss of functional capacity after ICU admission may be a determining factor for eligibility for anticancer therapies.

## CONCLUSION

There are “positive deviances” in the care of critically ill patients with cancer that have the potential to generate high-value care from patient admission to end-of-life care considering the experiences of patients and their families.

The implementation of such policies is feasible for most centers that care for this population. Even for facilities with scarce resources, applying resources that add value is imperative.

Initiatives such as this paper are one of the first steps in raising awareness and beginning a discussion about high-value care in various health scenarios.
